# APPLICABILITY OF A GENERIC QUESTIONNAIRE FOR QUALITY OF LIFE ASSESSMENT FOR ASTHMATIC CHILDREN

**DOI:** 10.1590/1984-0462/;2018;36;2;00006

**Published:** 2018-03-29

**Authors:** Ana Aline Marcelino da Silva, Álvaro Campos Cavalcanti Maciel, Priscilla Rique Furtado, Renata Ramos Tomaz, Thalita Medeiros Fernandes de Macêdo, Karla Morganna Pereira Pinto de Mendonça

**Affiliations:** a Universidade Federal do Rio Grande do Norte, Natal, RN, Brasil.

**Keywords:** Asthma, Children, Quality of life, Asma, Crianças, Qualidade de vida

## Abstract

**Objective:**

To determine the applicability of the generic instrument Pediatric Quality of Life Inventory (PedsQL 4.0) to assess health-related quality of life of asthmatic children compared to the specific instrument Pediatric Asthma Quality of Life Questionnaire (PAQLQ).

**Methods:**

This research involved the evaluation of 30 children aged seven to eleven years, who had been diagnosed with asthma for at least six months prior to research. Both quality of life questionnaires were applied to children by an interviewer over the period of one day. Data were analyzed using the Statistical Package for the Social Sciences (SPSS) 20.0, with significance level set at 5%.

**Results:**

No differences in quality of life was found when genders were compared. Asthmatic children classified as practitioners and non-practitioners of physical activity had different scores in the physical health-related domain (PedsQL 4.0). The scores of children with different levels of asthma severity and control were significantly different in two out of three domains evaluated by PAQLQ. When assessed by PedsQL 4.0, no significant difference was observed as to quality of life of children with different levels of asthma control and severity. Quality of life measurements of asthmatic children by PedsQL 4.0 and PAQLQ instruments had a moderate and significant correlation (r=0.415, p=0.02).

**Conclusions:**

PedsQL 4.0 could distinguish children practicing different levels of physical activity, but it was not sensitive enough to distinguish health-related quality of life among children with different levels of asthma severity and control.

## RESUMO

**Objetivo:**

Avaliar se o instrumento genérico *Pediatric Quality of Life Inventory* (PedsQL 4.0) é aplicável à avaliação da qualidade de vida relacionada à saúde (QVRS) de crianças asmáticas, quando comparado ao instrumento específico *Pediatric Asthma Quality of Life Questionnaire* (PAQLQ).

**Métodos:**

Foram avaliadas 30 crianças com idade entre sete e onze anos de idade e diagnóstico de asma havia, pelo menos, seis meses. Ambos os questionários foram respondidos no mesmo dia, por meio de entrevista. Os dados foram analisados com o auxílio do *Statistical Package for the Social Sciences* (SPSS), versão 20.0, atribuindo-se o nível de significância de 5%.

**Resultados:**

Não ocorreram diferenças significativas na qualidade de vida das crianças avaliadas de acordo os sexos. As crianças asmáticas classificadas como praticantes e não praticantes de atividade física obtiveram escores significativamente diferentes no domínio relacionado à saúde física (PedsQL 4.0). Crianças com diferentes níveis de gravidade e controle da asma apresentaram diferenças significativas nos escores obtidos em dois dos três domínios avaliados por meio do PAQLQ. Ao serem avaliadas com o instrumento PedsQL 4.0, nenhuma diferença significativa foi observada na qualidade de vida das crianças com diferentes níveis de controle e gravidade da asma. As medidas de qualidade de vida de crianças asmáticas obtidas pelos instrumentos PedsQL 4.0 e PAQLQ apresentaram correlação moderada e significativa (r=0,415, p=0,02).

**Conclusões:**

O instrumento PedsQL 4.0 diferenciou as crianças com diferentes níveis de atividade física. No entanto, ele não foi sensível para diferenciar a qualidade de vida relacionada à saúde em crianças com diferentes níveis de gravidade e controle da asma.

## INTRODUCTION

The World Health Organization (WHO) defines quality of life as an individual’s perception of their position in life considering the context of culture and value systems in which they live and regarding their goals, expectations, standards and concerns.^1^ In addition to the generic term “quality of life”, there is also the notion of health-related quality of life (HRQoL). This word is very recurrent in the literature and has been used with objectives similar to the more general concept. However, it applies to aspects more directly related to diseases or health interventions.^2^

The importance of assessing quality of life of children and adolescents, whether they are healthy or suffer from a chronic illness, is increasing since the technological evolution of medicine contributes to increased survival rates.^3^ Asthma, for example, is a chronic inflammatory disease that makes the airways hyper responsive and limits airflow due to bronchoconstriction, mucous blockage, and increased inflammation caused by exposure to certain risk factors. This disease has the greatest morbidity and mortality rates worldwide, and is manifested clinically by recurrent episodes of wheezing, dyspnea, chest tightness, and cough, usually at night and in the morning on waking up.^4^ Asthma accounts for 350,000 hospitalizations per year in the Brazilian public health system (*Sistema Único de Saúde* - SUS).^5^

The Pediatric Asthma Quality of Life Questionnaire (PAQLQ) is a specific questionnaire, used to evaluate HRQoL of asthmatic children and adolescents aged 7 to 17 years, which can be self-administered or answered in an interview.^6^ It was originally developed in English by Juniper et al.^7^ and later translated and culturally adapted to be applied to Brazilian children and adolescents.^8^ In 2007, Icaza^9^ evaluated the psychometric properties and validated the Brazilian version of PAQLQ with a sample of Brazilian children.

The Pediatric Quality of Life Inventory (PedsQL) is a generic questionnaire developed to evaluate HRQoL of children and adolescents aged 2 to 18 years who are healthy and bear chronic or acute health conditions, without specification as to type of condition;^10^ it was also conceived in English language by Varni in 1987, with successive new versions and items added until it became PedsQL 4.0. This questionnaire was validated for Brazil in a study carried out by Klatchoian *et al*. in 2008, with children and adolescents living in São Paulo.^11^

Grounded on the need of instruments easily applicable in clinical practice, the present study aimed to compare the measures obtained in the evaluation of quality of life of asthmatic children through the application of a generic (PedsQL4.0) and a specific questionnaire (PAQLQ), both developed and validated for this purpose.

## METHOD

The study included 30 asthmatic children aged between 7 and 12 years old. The diagnosis of asthma was made by a pulmonologist, based on children’s clinical history and symptoms presented and according to the criteria recommended by the Global Initiative for Asthma (GINA).^4^ Participating children were being assisted at the outpatient clinics of two pediatric reference hospitals in the city of Natal. They were supposed to be clinically stable and could not present with any diagnosed heart, neuromuscular, rheumatic, musculoskeletal or orthopedic diseases, and associated neurological sequelae that could expressively compromise quality of life. They were also not supposed to have had upper or lower respiratory infections in the three weeks prior to evaluations.^12^ ^,^ ^13^

The project was submitted to the Research Ethics Committee of *Universidade Federal do Rio Grande do Norte* (UFRN), in accordance with Resolution 466/12 of the National Health Council, under approval 876,304. Before the children answered the questionnaires, their parents or caregivers were requested to sign the informed consent form. Children who were unable to understand the questionnaires or decided to not participate in the evaluation were excluded from the study.

The evaluation was made at the UFRN Physiotherapy Department over one day. Initially, individual records were filled in with data such as name, age, sex, weight, height, sports practice or regular physical activity, asthma diagnosis, severity grading (intermittent, mild persistent, moderate persistent, severe persistent),^4^ ^,^ ^5^ asthma control (controlled, partially controlled, uncontrolled) according to the Brazilian Society of Pneumology and Tisiology (SBPT).^5^ Practice of physical activity was considered regular when children reported doing it for at least 30 minutes, three times a week.^1^ All participants then answered the two questionnaires in an interview conducted by the same previously trained interviewer. The order of application was random.

PAQLQ has 23 items subdivided into three domains: Activity Limitation (d-LimA, five items), Symptoms (d-Sint, ten items), and Emotional Function (d-Emo, eight items). Three items of d-LimA are adapted for each patient, that is, before starting the questionnaire, each child chooses the three activities of their daily life that they consider most compromised by asthma. All items in PAQLQ are similarly answered using a seven-point Likert scale, where 1 represents the highest degree of impairment (extremely impaired/all the time), and 7, no impairment (not impaired/never). The questionnaire assigned scores in total and by domains based on the average scores of corresponding items, and all questions are related to the respondents’ previous week. The minimum significant difference (MSD) between evaluations is 0.5 point.^9^

PedsQL 4.0 features three versions for children and three equivalents, with similar questions, for their respective caregivers. The versions were designed to suit cognitive levels: 5-7 years, 8-12 years and >12 years. The version of the instrument for eight+ year-olds has 23 items divided into four domains: physical (d-Fis, eight items), emotional (d-Emoc, five items), social (d-Soc, five items), and school function (d-Esco, five items). A fifth domain - psychosocial (d-Psycho) - is the sum of the former three. Each item has five Likert scale response options (never: 0 almost always: 4), values later operationalized and transformed into a reverse linear scale from 0 to 100, where the highest score represents the best state. Questions are asked regarding the past month of respondents. The questionnaire is scored in total and by dimensions, based on mean scores of corresponding items.^9^ The version used in the present study was 8-12 years, for the questionnaires intended for the evaluation of children aged 5 to 7 years and 8 to 12 years are essentially similar, differing only in terms of language to best suit the level of cognitive development.^11^ The language was adapted by the interviewer for proper understanding by 7-year-olds, as suggested by the instrument itself.

The sample data were analyzed using SPSS 20.0 (Statistical Package for the Social Sciences, Chicago, USA) for Windows^®^. The Kolmogorov-Smirnov test was used to verify data normality. In the descriptive analysis, quantitative variables were shown as mean and standard deviation (SD), while categorical data were expressed as frequencies. Then, the unpaired Student t test and the analysis of variance (ANOVA) were used to compare the means of continuous independent variables according to groups and depending on their nature. Pearson’s correlation (r) was also performed to analyze the behavior between quantitative variables, which were the domains of both questionnaires. Throughout the analysis, p-value of 0.05 and 95% confidence interval (95%CI) were considered. The power was 0.87, calculated afterwards to compare measures obtained in the quality of life evaluation of both questionnaires.

## RESULTS

The study included 30 children, 12 females (40.0%) and 18 males (60.0%), mean age 9.0±1.7 years, mean weight 33.8±8.2 kg and mean height 1.3±0.1 m. Regarding asthma severity: 20.0% had intermittent asthma; 30.0%, mild persistent; 33.3%, moderate persistent; and 16.7%, severe. Asthma control was classified as controlled (40.0% of subjects), partially controlled (50.0%), and uncontrolled (10.0%). Most children (83.3%) did not practice physical activity.

The scores obtained in PAQLQ and PedsQL 4.0 for each domain are presented below as mean and DP. 

PAQLQ scores were:

- Physical activity limitation score (4.8±1.3);
- Symptoms score (5.4±1.5);
- Emotional function score (5.6±1.4);
- Total score (5.3±1.3).

PedsQL 4.0 scores were:

- Physical health score (72.3±18.2);
- Psychosocial health score (70.7±14.8);
- Total score (71.2±13.8).

The first comparative analysis between scores of both questionnaires and genders showed no significant differences. Table 1 shows the comparison between scores of questionnaires and the practice of sports or physical activity. A significant difference between practitioners and non-practitioners of sports as to scores for the physical health domain of PedsQL 4.0 can be noted.

**Table 1:** Comparison between scores of Pediatric Asthma Quality of Life Questionnaire and Pediatric Quality of Life Inventory for practice of physical activity/sports by children interviewed.

	Practice of Physical activity/ sports Y (n=5)/ N (n=25)	Questionnaires’ scores (M±SD)	p-value
PAQLQ			
Physical activity limitation score	Y	4.9±0.7	0.82
	N	4.8±1.4	
Symptoms score	Y	5.5±0.9	0.89
	N	5.4±1.5	
Emotional function score	Y	5.8±0.5	0.59
	N	5.5±1.4	
Total score	Y	5.5±0.5	0.76
	N	5.3±1.4	
PedsQL			
Physical health score	Y	83.1±6.4	0.01*
	N	70.1±19.1	
Psychosocial health score	Y	70.9±13.3	0.95
	N	70.5±15.3	
Total score	Y	75.2±10.6	0.48
	N	70.4±14.3	

Y: yes; N: no; M: mean; SD: Standard deviation; *p<0.05; PAQLQ: Pediatric Asthma Quality of Life Questionnaire; PedsQL: Pediatric Quality of Life Inventory.

Table 2 displays the comparison between domains of PAQLQ and PedsQL 4.0 according to asthma severity grading in children evaluated. For statistical purposes, the sample was classified into three categories: intermittent, mild persistent/moderate, and severe persistent. Comparison between asthma control and questionnaire scores was also performed. For statistical purposes, the control variable was divided as controlled and uncontrolled asthma. The results of this comparison are described in Table 3.

**Table 2:** Comparison between scores of Pediatric Asthma Quality of Life Questionnaire and Pediatric Quality of Life Inventory for asthma severity grading of children interviewed.

	Asthma severity I (n=6), MP/M (n=19) SP (n=5)	Questionnaires’ scores (M±SD)	p-value
PAQLQ			
Physical activity limitation	I	5.7±0.5	0.03*
	MP/M	4.8±1.4	
	SP	3.6±0.7	
	Total	4.8±1.3	
Symptoms	I	6.0±0.7	0.01*
	MP/M	5.6±1.2	
	SP	3.7±1.8	
	Total	5.4±1.4	
Emotional function	I	6.2±0.7	0.21
	MP/M	5.5±1.4	
	SP	4.7±1.5	
	Total	5.5±1.3	
Total	I	6.0±0.2	0.03*
	MP/M	5.4±1.2	
	SP	4.0±1.4	
	Total	5.3±1.2	
PedsQL			
Physical health	I	71.3±28.6	0.79
	MP/M	73.8±15.7	
	SP	67.4±15.2	
	Total	72.2±18.2	
Psychosocial health	I	76.3±14.7	0.23
	MP/M	71.3±15.0	
	SP	61.3±11.6	
	Total	70.6±14.8	
Total	I	74.6±15.2	0.36
	MP/M	72.1±14.3	
	SP	63.4±7.3	
	Total	71.2±13.7	

I: intermittent; MP/M: mild persistent/moderate; SP: severe persistent; M: mean; SD: Standard deviation; *p<0.05; PAQLQ: Pediatric Asthma Quality of Life Questionnaire; PedsQL: Pediatric Quality of Life Inventory.

**Table 3:** Comparison between scores of Pediatric Asthma Quality of Life Questionnaire and Pediatric Quality of Life Inventory for asthma control of children interviewed.

	Asthma control C (n=12)/UC (n=18)	Questionnaires’ scores (M±SD)	p-value
PAQLQ			
Physical activity limitation	C	5.2±1.0	0.16
	UC	4.5±1.4	
Symptoms	C	6.1±0.8	0.03*
	UC	4.9±1.6	
Emotional function	C	6.4±0.5	0.007*
	UC	5.0±1.4	
Total	C	6.0±0.6	0.006*
	UC	4.9±1.4	
PedsQL			
Physical health	C	73.6±20.8	0.16
	UC	71.4±16.8	
Psychosocial health	C	75.2±12.1	0.16
	UC	67.5±15.9	
Total	C	74.7±12.3	0.26
	UC	68.8±14.4	

C: controlled; UC: uncontrolled; M: mean; SD: Standard deviation; *p<0.05; PAQLQ: Pediatric Asthma Quality of Life Questionnaire; PedsQL: Pediatric Quality of Life Inventory.

When correlating PAQLQ and PedsQL 4.0 scores, a moderate correlation (greater than 0.40) was found in only two domains of both questionnaires, as described in Table 4.

**Table 4:** Correlation between scores of Pediatric Asthma Quality of Life Questionnaire e do Pediatric Quality of Life Inventory.

PedsQL Score	Physical health	Psychosocial health	Total
PAQLQ Score			
Physical activity limitation	r=0.26 (p=0.17)	r=0.29 (p=0.11)	-
Symptoms	r=0.25 (p=0.19)	r=0.37 (p=0.04)	-
Emotional function	r=0.13 (p=0.50)	r=0.49 (p=0.006)	-
Total	-	-	r=0.41(p=0.02)

r: Pearson’s correlation coefficient; PAQLQ: Pediatric Asthma Quality of Life Questionnaire; PedsQL: Pediatric Quality of Life Inventory.

## DISCUSSION

The findings of this study show that PedsQL 4.0, through scores obtained in physical health domain (d-Fis, eight items) distinguished practice and non-practice of physical activity. Only the specific questionnaire for HRQoL of asthmatic children (PAQLQ) showed variances between children with different levels of asthma severity and control. Children with different severity levels presented different scores in domains d-LimA (five items), d-Sint (ten items) and total PAQLQ score. This same instrument identified children with different levels of asthma control by scores obtained in symptoms domain, d-Emo (eight items), and, similarly, in total score. Between generic and specific instruments PedsQL 4.0 and PAQLQ, respectively, a moderate correlation (> 0.40) was found between emotional function domains of PAQLQ and psychosocial health domain of PedsQL, as well as between total scores of both instruments.

When evaluating Brazilian children living in Rio Grande do Sul, Icaza^9^ found mean values similar to ours for PAQLQ. However, in a study with children and adolescents from Portugal, Guedes^14^ found higher mean values for the same questionnaire. When it comes to PedsQL 4.0 questionnaire, both Icaza^9^ and Guedes^14^ found higher mean values compared to the present study. The higher mean values obtained in other studies may be explained by factors influencing HRQoL in different populations. Socioeconomic factors such as parents’ higher educational level and better family socioeconomic status are reported to positively influence quality of life in the literature.^15^

In the present study, a higher total PAQLQ score (6.0) was found for children with intermittent asthma and a lower score (4.0) for those with severe persistent asthma. This is in line with literature data, which indicates that, although the questionnaires are not fully related to disease control, children with severe asthma tend to have a poorer HRQL compared to those with mild to severe levels.^16^ ^,^ ^17^ For PedsQL, no significant difference was found between scores and asthma severity level.

The lack of differences between questionnaires and individuals’ gender in the present study is corroborated by previous investigations. Roncada^17^ and Nogueira et al.,^18^ also did not find significant differences regarding gender when evaluating quality of life of asthmatic children and adolescents. In contrast, Rydström et al.^19^ and Zandieh et al.^20^ found differences in quality of life according to gender in asthmatic children. The former^19^ pointed a lower quality of life among female children in Sweden, while the latter^20^ pointed low quality of life among males when evaluating Iranian children and adolescents aged between 7 and 17 years.

PedsQL’s d-Fis (eight items) showed better quality of life for children practicing physical activity compared to non-practitioners. The other PedsQL and PAQLQ scores did not differ. These findings corroborate a study previously developed by Nogueira et al.,^18^ in which no differences were found between practitioners and non-practitioners’ quality of life. However, when using the PAQLQ and PedsQL 4.0 instruments, Guedes^14^ pointed out significant differences between children/adolescents who would practice a sport or physical activity with both instruments. The author did not compare domains, though, having considered only total scores in the analysis.

As the mean scores of individuals of the present sample with mild and moderate asthma were very similar, the severity of the disease was divided into intermittent, mild persistent/moderate and severe persistent. Thus, significant differences were found between PAQLQ scores and asthma severity levels. However, the emotional function score showed no significant difference, like in the studies by Vidal et al.^21^ and Perosa et al.^16^ These authors report that results related to emotional domains are controversial, since there are studies in which children show fear, anxiety, and depression, while others bring no records of emotional losses, especially in younger children or those living with the disease for longer periods. For statistical purposes, asthma control was sorted as controlled and uncontrolled. Thus, when comparing scores of questionnaires as to asthma control, PAQLQ had statistically significant differences for all scores, except physical activity limitation score. This can be explained by overprotection by mothers in the sample evaluated, in the sense of not allowing the child to perform certain activities or practice sports. However, PedsQL 4.0 scores were not significantly different as to asthma control, which shows that this questionnaire was not sensitive enough to differentiate quality of life of children with controlled asthma from that of children with uncontrolled disease.

Correlation between total scores of PAQLQ and PedsQL 4.0 was moderate. We also found a moderate correlation between PAQLQ’s emotional function score and PedsQL’s psychosocial health score, since the psychosocial health score also includes emotional functioning. All other scores wwere found to have weak correlations between them.

Our findings are in accordance with Guedes’;^14^ the author performed a concordance analysis between both questionnaires and pointed that PedsQL 4.0 scores more in comparison to PAQLQ in situations of lower quality of life, which shows that PedsQL 4.0 ignores some aspects detected by PAQLQ. Thus, PAQLQ is specific for children and adolescents with asthma, in addition to detecting subtle changes in the quality of life of this group of patients.

One of the problems faced while assessing quality of life was the lack of own instruments or locally and culturally adapted versions of existing ones.^9^ Thus, the present study aimed to evaluate the applicability of a questionnaire and verify the possibility of using more than one easy-application and understanding tool in the age range it was elaborated for. A few aspects may be cited as possible limitations of the study, though, such as the disregarding of socioeconomic factors in the evaluation (since they could interfere in the HRQoL of children evaluated) and the small sample size.

Among the main findings of this study, we can highlight the fact that PAQLQ and PedsQL 4.0 questionnaires are significantly correlated as to total scores and, as to domains, between the emotional function scores of PAQLQ and psychosocial health of PedsQL; besides, only the physical health domain of PedsQL could distinguish children who did or did not practice physical activity. From the comparisons between questionnaires, conclusion is that PedsQL 4.0 was not sensitive in distinguishing quality of life between asthmatic children with different levels of severity and control of the disease, since domains were not specific for this purpose.
